# Septic shock caused by *Rhizobium radiobacter* in an elderly woman: A case report

**DOI:** 10.1097/MD.0000000000018267

**Published:** 2019-12-10

**Authors:** Dong-lian Wang, Li-dong Zhao, Li-juan Li, Min-jie Zhou

**Affiliations:** Department of Emergency Medicine, Shanghai Sixth People's Hospital, Shanghai Jiao Tong University School of Medicine, Shanghai, China.

**Keywords:** infection, *Rhizobium radiobacter*, sepsis

## Abstract

**Rationale:**

*Rhizobium radiobacter* is a Gram-negative pathogen present in soil and plants. Cases of *R radiobacter* infection in immunocompromised hosts have been sporadically reported. However, septic shock caused by *R radiobacter* is rarely seen.

**Patient concerns:**

Here, we describe an elderly patient with a rapid progression of watery diarrhea, anorexia, fever, weakness, oliguria, and shock. Blood results showed increased total white blood cell count and C-reactive protein. Arterial blood gas results showed hypoxia and elevated lactate level. The Sequential Organ Failure Assessment score was 11. Blood culture at admission showed Gram-negative bacteria, which were later confirmed as *R radiobacter*.

**Diagnosis:**

Septic shock caused by *R Radiobacter.*

**Interventions:**

The patient was treated with intravenous cefoperazone/sulbactam and sequential oral levofloxacin.

**Outcomes:**

The patient recovered completely.

**Conclusion:**

*R radiobacter* may be considered as a potential opportunistic pathogen that may cause severe sepsis in elderly patients, especially those with multiple underlying diseases.

## 1 Introduction

*Rhizobium radiobacter* is a Gram-negative pathogen that is found in plants and soil.^[1]^*R radiobacter* infection in humans is very rare.^[2,3]^ Sporadic cases of *R radiobacter* infection have been reported in immunocompromised hosts and those with indwelling catheters. The prognosis is generally good owing to its low virulence. Here we present a case of *R radiobacter* infection in an elderly patient with multiple comorbidities. The patient rapidly progressed to septic shock but eventually showed complete recovery after prompt antibiotic treatment.

## 2 Case report

An 87-year-old woman was admitted to the emergency department with a 2-day-long history of watery diarrhea, anorexia, fever, progressive weakness, and oliguria. The symptoms presented after taking oral laxatives due to persistent constipation. The patient had a history of cerebral infarction, hypertension, and coronary heart disease which were all well-controlled; the patient had no stroke sequelae. At admission, she was lethargic and apathetic. Her vital signs were: temperature, 38.9°C; heart rate, 126 beats per minute; blood pressure, 67/53 mmHg. Her breath sounds were rough; however, no dry or wet crackles were heard. Cardiac auscultation showed no heart murmur. A scaphoid abdomen was noticed and the bowel sounds were audible. Other physical findings were unremarkable. Blood results showed elevated total white blood cell count (17.33 × 10^9^ cells/L; neutrophils, 81.9%) and increased level of C-reactive protein (137.45 mg/L). Arterial blood gas results were: pH 7.53; PCO_2_ 26 mmHg; PO_2_ 64 mmHg; lactate 2.3 mmol/L. Serum potassium level was slightly decreased (3.3 mmol/L), whereas serum creatinine (116 μmol/L) was elevated. A diagnosis of septic shock was established (Sequential Organ Failure Assessment [SOFA] score: 11)^[4]^ (Table 1). Prompt antibiotic therapy with cefoperazone/sulbactam (3.0 g q8 h; Pfizer, W62921) was initiated along with fluid resuscitation and vasoactive support. Aerobic blood sample showed Gram-negative bacteria the next day after incubation. Bacteria were transferred onto a sheep blood agar plate and cultured in a 5% CO_2_ incubator. The isolate was identified as *R radiobacter* by VITEK-2 (fully automated identification system). Owing to the lack of established criteria to determine drug sensitivity of *R radiobacter*, inhibition zone diameter was determined using the disk diffusion method. The results are shown in Table 2. Empirical antibiotic therapy with cefoperazone/sulbactam was consistent with the results of drug sensitivity. The patient responded to treatment and was discharged with stable vital signs after 10 days of treatment. Pre-discharge blood culture results were normal. At discharge, the patient was prescribed oral levofloxacin for 1 week and she fully recovered thereafter.

**Table 1:**
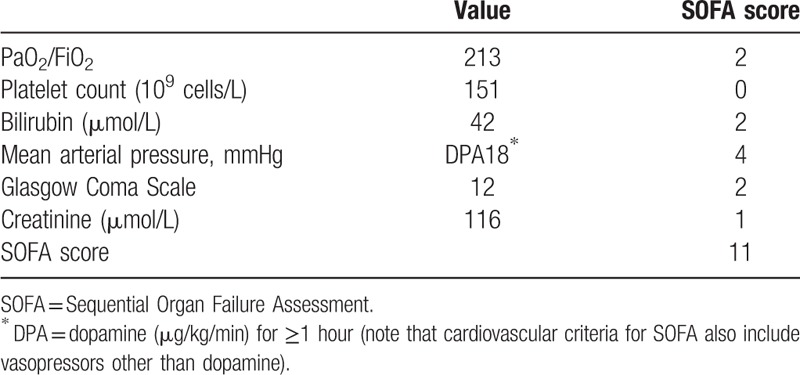
Calculation of SOFA score.

**Table 2:**
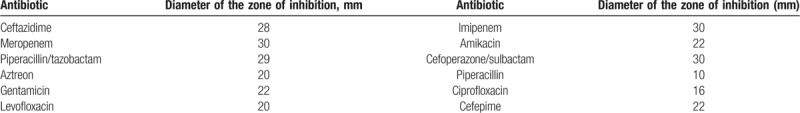
The inhibition zone diameter determined by disk diffusion method.

## 3 Discussion

The genus *Rhizobium* (formerly *Agrobacterium*) includes pathogens of agriculture soil and plants, which are usually associated with plant tumorigenic diseases.^[5]^ Several species of *Rhizobium* have been identified so far; these include, *R radiobacter*, *R rhizogenes*, *R rubi*, *R undicola,* and *R vitis*. Among these, *R radiobacter* is the most commonly reported opportunistic pathogen in humans.^[6]^

Infection caused by *R radiobacter* is typically community-acquired and affects immunodeficient or chronically debilitated hosts with underlying conditions such as human immunodeficiency virus (HIV) infection, malignancies, bone marrow transplant recipients, chronic renal failure with dialysis, diabetes, and those receiving corticosteroid therapies.^[2,7,8]^ In this case, the patient was in old age and had multiple underlying diseases such as cerebral infarction, hypertension, and coronary heart disease. In addition, there was a recent history of soil exposure, which likely led to the infection.

*R radiobacter* infection in humans have included urinary tract infection, and rarely bacteremia, endocarditis, endophthalmitis, peritonitis, brain abscess, pneumonia, and spondylodiscitis.^[8–12]^ Catheter- or plastic surgery-related *R radiobacter* infection has been the most commonly reported presentation.^[2,3,7,13]^ In early summaries of *R radiobacter* infection, the majority (77%) were directly associated with foreign bodies.^[2,14]^

*R radiobacter* has been reported as a potential pathogen in pediatric patients because of the underdeveloped immune system. The reported frequency of *R radiobacter* infection in pediatric patients with in-dwelling catheter is approximately 2.56%.^[15]^ However, due to the low incidence, there has been no large-scale epidemiological investigation of the incidence of *R radiobacter* in adults; the published literature largely pertains to sporadic case reports. In our patient, no catheter or plastic surgery was involved. The infection likely originated from the digestive system with symptoms of diarrhea and anorexia. The condition rapidly progressed to septic shock. Of note, no mortality has been attributed directly to *R radiobacter* infection in previously reported cases.^[2,13]^ However, our patient had a SOFA score of 11 and the estimated risk of mortality was >50%, which implies that *R radiobacter* infection may be potentially fatal, especially in elderly patients with multiple comorbidities.^[16]^

The optimal antibiotic treatment for *R radiobacter* infection has not been determined due to its low incidence. Our antimicrobial susceptibility results showed that *R radiobacter* was sensitive to third-generation cephalosporins, aminoglycosides, fluroquinolones, and carbapenems, which is consistent with previous reports.^[2,13,17]^ Hence, we selected cefoperazone/sulbactam as the initial empirical therapy and successfully eliminated *R radiobacter* infection. Previous studies showed that *R radiobacter* may be resistant to aminoglycosides, including gentamicin.^[2,18]^ Therefore, aminoglycosides are not recommended for empirical treatment of *R radiobacter* infection. In patients with in-dwelling catheter or foreign body, removal of the catheter or implanted medical device is an important treatment strategy.^[1]^ In this case, the duration of treatment was approximately 2 to 3 weeks; this is in line with previous studies that suggested 10 to 14 blood culture-sterile days as the optimal duration of treatment.^[3]^

## 4 Conclusions

*R radiobacter* is an opportunistic Gram-negative pathogen that mainly affects immunocompromised children and adults, especially those with in-dwelling catheters or plastic implants. However, it may occur in elderly patients with multiple comorbidities. *R radiobacter* is sensitive to third-generation cefoperazone/sulbactam and fluroquinolones. Although it typically exhibits low virulence, it may cause septic shock and may be potentially fatal in the absence of prompt treatment.

## Author contributions

**Conceptualization:** Dong-lian Wang, Li-dong Zhao.

**Data curation:** Dong-lian Wang, Li-dong Zhao, Li-juan Li.

**Formal analysis:** Dong-lian Wang, Li-juan Li, Min-jie Zhou.

**Funding acquisition:** Dong-lian Wang, Min-jie Zhou.

**Investigation:** Min-jie Zhou.

**Supervision:** Li-dong Zhao, Li-juan Li.

**Visualization:** Dong-lian Wang.
